# Non-invasive determination by cardiovascular magnetic resonance of right ventricular-vascular coupling in children and adolescents with pulmonary hypertension

**DOI:** 10.1186/s12968-015-0186-1

**Published:** 2015-09-16

**Authors:** Uyen Truong, Sonali Patel, Vitaly Kheyfets, Jamie Dunning, Brian Fonseca, Alex J. Barker, Dunbar Ivy, Robin Shandas, Kendall Hunter

**Affiliations:** Division of Pediatric Cardiology, Children’s Hospital Colorado, Aurora, CO 80045 USA; Department of Bioengineering, University of Colorado Denver Medical Campus, Aurora, CO 80045 USA; Department of Radiology, Northwestern University, Chicago, IL USA; Department for Pediatrics, Division of Cardiology, Children’s Hospital Colorado, University of Colorado Anschultz Medical Center, 13123 E. 16th Avenue, B100, Aurora, CO 80045 USA

**Keywords:** Ventricular vascular coupling ratio, Non-invasive, Pediatrics, Pulmonary hypertension, Cardiovascular magnetic resonance

## Abstract

**Background:**

Pediatric pulmonary hypertension (PH) remains a disease with high morbidity and mortality in children. Understanding ventricular-vascular coupling, a measure of how well matched the ventricular and vascular function are, may elucidate pathway leading to right heart failure. Ventricular vascular coupling ratio (VVCR), comprised of effective elastance (E_a_, index of arterial load) and right ventricular maximal end-systolic elastance (E_es_, index of contractility), is conventionally determined by catheterization. Here, we apply a non-invasive approach to determining VVCR in pediatric subjects with PH.

**Methods:**

This retrospective study included PH subjects who had a cardiovascular magnetic resonance (CMR) study within 14 days of cardiac catheterization. PH was defined as mean pulmonary artery pressure (mPAP) ≥ 25 mmHg on prior or current catheterization. A non-invasive measure of VVCR was derived from CMR-only (VVCR_m_) and compared to VVCR estimated by catheterization-derived single beat estimation (VVCR_s_). Indexed pulmonary vascular resistance (PVRi) and pulmonary vascular reactivity were determined during the catheterization procedure. Pearson correlation coefficients were calculated between PVRi and VVCR_m_. Receiver operating characteristic (ROC) curve analysis determined the diagnostic value of VVCR_m_ in predicting vascular reactivity.

**Results:**

Seventeen subjects (3 months-23 years; mean 11.3 ± 7.4 years) were identified between January 2009-August 2013 for inclusion with equal gender distributions. Mean mPAP was 35 mmHg ± 15 and PVRi was 8.5 Woods unit x m2 ± 7.8. VVCR_m_ (range 0.43–2.82) increased with increasing severity as defined by PVRi (p < 0.001), and was highly correlated with PVRi (r = 0.92, 95 % CI 0.79–0.97, p < 0.0001). Regression of VVCR_m_ and PVRi demonstrated differing lines when separated by reactivity. VVCR_m_ was significantly correlated with VVCR_s_ (r = 0.79, CI 0.48–0.99, p <0.0001). ROC curve analysis showed high accuracy of VVCR_m_ in determining vascular reactivity (VVCR = 0.85 had a sensitivity of 100 % and a specificity of 80 %) with an area under the curve of 0.89 (*p* = 0.008).

**Conclusion:**

Measurement of VVCR_m_ in pediatrics is feasible. Pulmonary vascular non-reactivity may be contribute to ventricular-vascular decoupling in severe PH. Therapeutic intervention to maintain a low vascular afterload in reactive patients may preserve right ventricular functional reserve and delay the onset of RV-PA decoupling. Use of VVCR_m_ may have significant prognostic implication.

## Background

Pulmonary hypertension (PH) in children remains an incurable disease with poor long-term prognosis that crosses all racial and socioeconomic backgrounds [1]. PH affects both adults and children, with an annual incidence of adult PH estimated to be 50 per million [2] and pediatric PH estimated to be 63 per million [3]. While there are some similarities between adult and pediatric pathophysiology [4], the disease may be more severe with worse survival in children [5–7]. This higher severity in youth is in due to differences in underlying causes, presence of congenital heart disease, effects of maturation, and differences in clinical symptoms leading to diagnosis in later stages [8]. Different from adult PH, the etiology of pediatric PH is mostly idiopathic pulmonary arterial hypertension and PH associated with congenital heart disease [1]. The main cause of mortality is failure of the right ventricle (RV), which is the result of multifactorial interactions including the increase of arterial resistance, pressure, and stiffness with the decoupling of the ventricular-vascular system. Yet, in clinical practice, pulmonary vascular resistance (PVR) is the primary focus for the clinicians. Recently, there has been increased interest in a more comprehensive understanding of the interaction of the RV and vasculature, and the role that this interaction plays in the pathophysiology of PH [9–12].

The ventricular-vascular coupling ratio (VVCR), the ratio of contractility to afterload, reflects the ventricular functional reserve in response to a rising afterload in order to maintain cardiac output. VVCR has been recently shown to be prognostic of impending RV failure [10]. Conventionally, VVCR is laboriously determined invasively, in the cardiac catheterization laboratory with the establishment of the pressure-volume loop under varying loading conditions and clamping of the inferior vena cava [6]. This can also be estimated via catheterization using a single beat method without altering preload or afterload, which has been validated for the left [13, 14] and right ventricle [15]. Recently, Sanz et al. [10] proposed using cardiovascular magnetic resonance (CMR) to estimate VVCR. The authors show that an estimation of VVCR is feasible solely with noninvasive volumetric data from CMR. Recently, Vanderpool et al. showed that this approach, applied to adults with PH, is a predictor of survival [12].

In this study, we apply Sanz’s approach to the pediatric population with PH to determine VVCR in the right heart and compare it to the modified single beat method. Determining the VVCR by noninvasive measures is critical in the pediatric population. The lack of exposure to radiation, anesthesia, and intracardiac catheter manipulations with CMR are important concerns to consider, as these patients often undergo multiple catheterization procedures throughout their lives to establish diagnosis and monitor responses to medications. As a result, the aims of this study are to: 1) determine if the noninvasive CMR approach to VVCR determination is feasible in a pediatric population, 2) understand how the technique compares with the more established single beat estimation method, and 3) determine the ability of the CMR-based VVCR method to predict patient outcome, as measured by acute vasoreactivity.

## Method

The medical records of children cared for in the Pulmonary Hypertension Program at Children’s Hospital Colorado were retrospectively reviewed from January 2009 to August 2013 for any patient, age 0 days to 65 years, who underwent a right heart catheterization (RHC) for pulmonary hypertension. The diagnosis of PH is defined by a previous or present cardiac catheterization showing mean pulmonary artery pressure (mPAP) ≥ 25 mmHg. Two hundred and forty-two patients were identified. Of these, we identified seventeen subjects who had a CMR study performed within 14 days of a cardiac catheterization. Exclusion criteria were: 1) pulmonary valve or pulmonary arterial stenosis; 2) previous surgical or catheterization intervention on the pulmonary valve or pulmonary artery; 3) pulmonary artery stenosis 4) previous right ventriculotomy; 5) congenital heart disease more complex than simple atrial septal defect; 6) more than mild pulmonary insufficiency; 7) severe left-sided obstruction that can potentially cause elevated pulmonary pressures; 8) chronic thrombolic pulmonary hypertension and 9) presence of arrhythmias. We excluded anatomic stenosis of the pulmonary circulation and chronic thrombolic PH because this is a study of vascular remodeling in the presence of high resistance rather than a study of the effects of anatomic constrictions or obstruction to flow. This study was carried out with the approval of the Colorado Institutional Review Board.

### Determination of VVCR

VVCR is composed of the arterial elastance (E_a_, representing afterload encountered by the ventricle and is a combination of resistance, compliance, and the time interval between systole and diastole) and the right maximal end-systolic elastance (E_es_, reflecting the index of ventricular contractility) [10]. In adults with PH, VVCR has been shown to be significantly increased [10]. Elevated VVCR reflects an inability of the RV function to compensate for an increasing pulmonary arterial load and thus results in mechanical inefficiency, leading to RV failure. This state is the early stages of ventricular-vascular decoupling, when cardiac mechanic efficiency declines in the face of a significant afterload.

#### VVCR by CMR

CMR studies were performed either on a 1.5 Tesla Siemens Avanto (Siemens Medical Solutions, Erlanger, Germany) or a 1.5 Tesla Philips Achieva (Philips Medical Systems, Best The Netherlands) scanner. Five of the youngest subjects were sedated for the CMR. Standard horizontal long axis and short axis images were obtained at end-expiration with a parallel stack of serial images acquired from the base to the apex [16]. Depending on the size of a subject, the parameters ranged from slice thickness of 4–10 mm, number of averages 1–3, TE 1.1–1.5, TR 2.8–3.5, and in-plane resolution 1.2–1.4 mm. Volumes were measured on Qmass software (MEDIS Medical Imaging Systems, Leiden, The Netherlands) by manually tracing the endocardial border at end diastole and end-systole. From this, data including end-systolic volume indexed to BSA, stroke volume indexed to BSA, and ejection fraction were calculated. Sanz’s approach was used to calculate $VVC{R}_m = \frac{ESV}{SV}$ [10]. RV ejection fraction is calculated as the difference in volume at end-diastole and end-systole, all over the end-diastolic volume × 100.

#### VVCR by single beat estimation

##### Right heart catheterization

All subjects were under general anesthesia for the RHC. A balloon-wedge catheter was introduced through the femoral vein or internal jugular vein and advanced into the right heart employing standard methodologies. Hemodynamic measurements were obtained, including right ventricular end-diastolic pressure, mPAP, transpulmonary gradient, and PCWP pressures. The Fick equation, with cardiac index calculated as cardiac output divided by body surface area, was used to determine cardiac output. PVR was calculated as the difference between mPAP and PCWP, all divided by the cardiac index. Indexed PVR is the product of PVR and body surface area. Part of the standard PH right heart catheterization at our institution is the determination of vascular reactivity with data obtained at room air and under vasodilator challenge. The vasodilator challenge involves 80–100 % oxygen or oxygen + inhaled nitric oxide (low dose: 20/40 ppm provided in the inhaled form). Vascular reactivity was determined by the Barst criteria [17], defined as

- Decrease in mPAP of ≥ 20 %,
- Unchanged or increased cardiac index, and
- Decreased or unchanged PVR to systemic vascular resistance ratio.

#### Single beat estimation

**Fig. 1:**
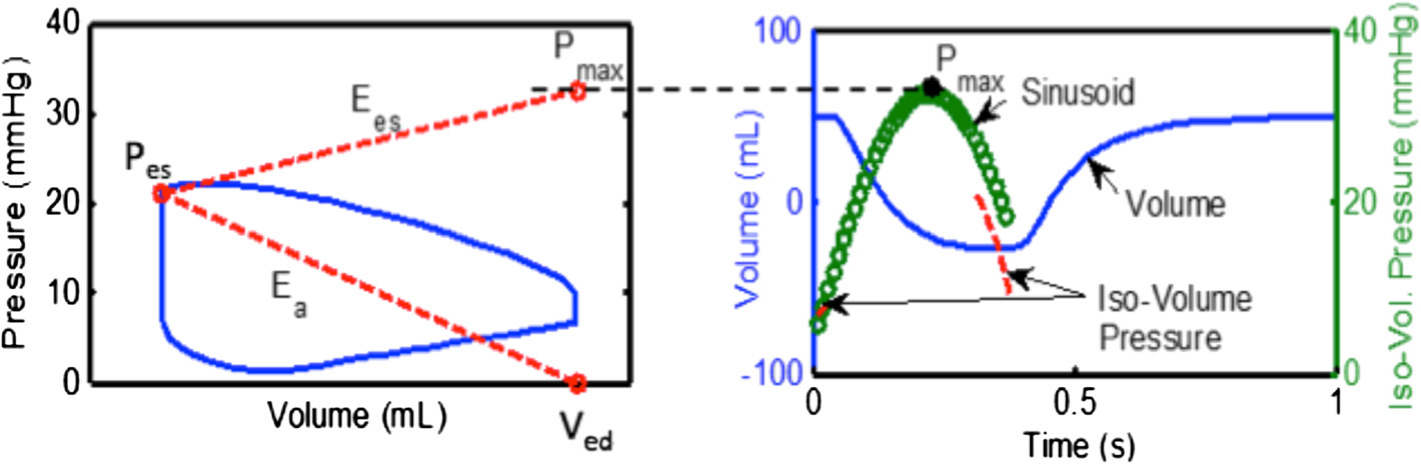
This diagram shows the single beat method by depicting how E_es_ and E_a_ are determined using the maximal pressure (P_max_) as the peak of the sinusoid of a ventricular pressure tracing, end-systolic point (P_es_), and (volume at end-diastole) V_ed_

Once RV pressure traces were successfully acquired, analysis was performed using a custom MATLAB (The Mathworks, Natick, MA) to yield VVCR_s_. The single beat method was first proposed by Takeuchi et al. [14] in which the authors proposed a novel method for the calculation E_es_ using the pressure tracing for a single beat. In this method, the theoretical maximum pressure the ventricle can generate is found by fitting a sinusoid to the iso-volumetric regions of the pressure tracing. The maximum of the sinusoid (Pmax) is used with the end-systolic ventricular pressure (Pes) to compute the slope of the end-systolic pressure volume relationship, Ees. (Fig. 1). This method has been validated for the right ventricle as well [15].

We modified this technique even further. Closely examining Fig. 1, it can be seen that

1 $${E}_{es}\cong \frac{P_{max}-{P}_{es}}{SV}.$$

Further given the definition [18, 19]

2 $${E}_a=\frac{P_{es}}{SV},$$

Thus, we can take the ratio of equations (1) and (2) to obtain VVCR_s_ as

3 $$\frac{E_a}{E_{es}}\cong \frac{SV}{P_{max}-{P}_{es}}.\frac{P_{es}}{SV}=\frac{P_{es}}{P_{max}-{P}_{es}}$$

Thus, equation (3) approximates VVCR_s_ as a simple ratio of the inverse of maximum isovolumic pressure to end-systolic pressure.

### Statistics

Statistical analysis was performed using the Statistical Analysis System (version 9.3; SAS Corporation, Cary, NC). Data were evaluated for normality using a Shapiro-Wilk test. Normally distributed, continuous data are presented as mean with standard deviation and categorical variables are presented as frequencies and percentages. Regression analysis was performed to estimate the relationship between VVCR_m_ and PVRi. In addition, Pearson correlation coefficients were calculated to determine strength of the relationship. Receiver-operating characteristic (ROC) curve analysis was used to determine the cut-off value of VVCR_m_ that was most frequently associated with pulmonary vascular reactivity, determined by the Barst criteria. This cut-off value maximizes the sum of sensitivity and specificity, which is equivalent to maximizing the difference between the sensitivity of the prognostic factor and the sensitivity that the prognostic factor would have if it did no better than random chance. Statistical significance was set at a p-value of 0.05 or less.

## Results

Seventeen subjects were identified for inclusion with equal gender distributions (Table 1). Age ranged from 3 months to years (mean 11.3 ± 7.4 years). The following diagnoses were noted for the subjects: 5 had idiopathic PH, 6 had PH associated with congenital heart disease, 1 associated with liver arteriovenous malformation, 1 associated with anthracycline, 1 associated with schistosomiasis, 2 with interstitial lung disease, and 1 with pulmonary hypertension associated with Overlap syndrome. Ten out of 17 subjects, who were old enough to have a World Health Organization (WHO) Functional Classification, were characterized by WHO class I or II. Six out of 17 subjects were on intravenous therapy. The National Institute of Health defines a child as an individual under 21 years of age.[20] We had only one subject above age 20 years. This particular subject was initially diagnosed at age 18 months with idiopathic PH and has been followed by our group since 13 years of age. Although the age at which he underwent the catheterization and CMR was as a young adult, his disease is consistent with childhood pulmonary hypertension. Eighty-two percent of the subjects had tricuspid regurgitation graded as none to mild based on echocardiograms performed within 5 days of the cardiac catheterization. Only one subject had severe tricuspid regurgitation.

**Table 1:** Demographic, hemodynamic, and cardiac magnetic resonance data for all subjects

Subject demographics (*n* = 17)	
Age	11.0 years (range 0.25–23.0)
Male gender	9/17
BSA (m^2^)	1.2 ± 0.5
Pulmonary Hypertension Classification^a^	
1. Pulmonary arterial hypertension	15
1.1 Idiopathic	5
1.3 Drug and Toxin-induced (anthracycline)	1
1.4.1 Connective tissue disorder (Overlap syndome)	1
1.4.3 Portal hypertension	1
1.4.4 Congenital heart disease	6
Atrial septal defect	4
Atrioventricular septal defect	1
Partial anomalous pulmonary venous return	1
1.4.5 Pulmonary hypertension from schistosomiasis	1
3. Pulmonary hypertension due to lung disease	2
World Health Organization Functional Classification	
WHO-FC I	2
WHO-FC II	8
WHO-FC III	4
WHO-FC IV	1
Pulmonary artery pressure	
Systolic pulmonary artery pressure (mmHg)	54.5 (20.6)
Diastolic pulmonary artery pressure (mmHg)	25.4 (11.7)
Mean pulmonary artery pressure (mmHg)	35 (15)
Pulmonary capillary wedge pressure (mmHg)	10.8 (3.2)
Pulmonary vascular resistance index (Woods unit x m2)	8.5 (7.8)
Cardiac output indexed (L/min/m2)	4.8 (1.5)
Right ventricular volume	
End-diastole (ml/m2)	118.4 (51.1)
End-systole (ml/m2)	70.9 (42.9)
Right ventricular ejection fraction (%)	46.6 (9.7)
Right ventricular stroke volume indexed (ml/m2)	54.4 (12.7)
E_a_ (mmHg/ml/m2)	0.49 (0.26)
E_max_ (mmHg/ml/m2)	0.56 (0.18)
VVCR_s_	1.79 (0.34)
VVCR_m_	1.29 (0.72)

^a^Pulmonary hypertension classification based on 5^th^ World Symposium in Nice, France in 2013 from Simommeau G et al. Updated clinical classification of pulmonary hypertension. JACC 2013:62:D34-41

### VVCR_m_ and disease severity

**Fig. 2:**
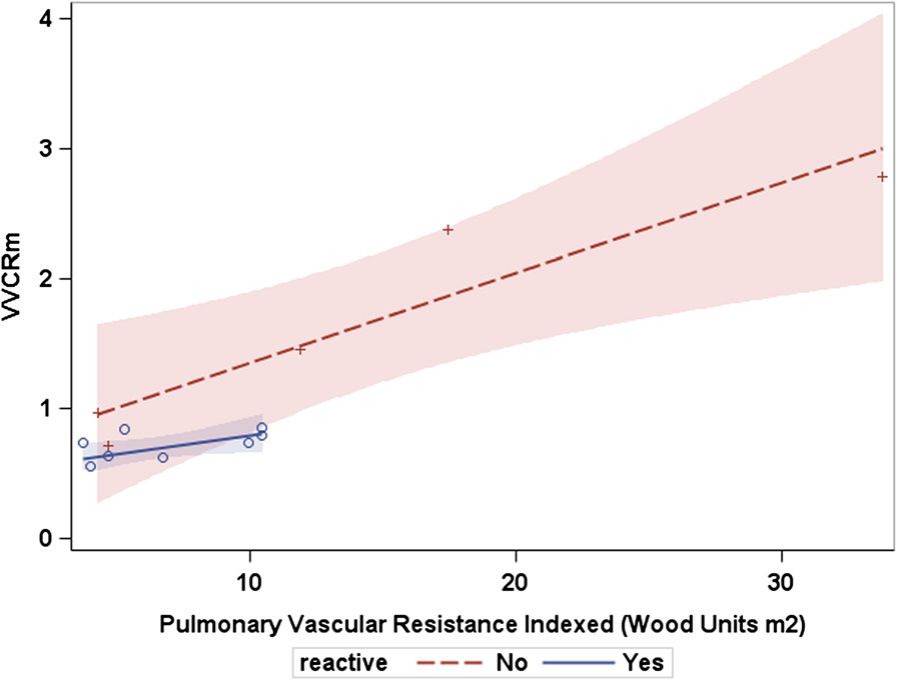
Regression of ventricular vascular coupling ratio derived by MRI (VVCRm) and pulmonary vascular resistance indexed by reactivity. The shaded areas represent the 95 % confidence interval for each regression line. The lines depict different trajectories based on reactivity, which approached, but not reach statistical significance (p > 0.05)

VVCR_m_ ranged from 0.84 to 3.29. While regression of VVCR_m_ and PVRi showed a linear relationship, a difference was seen in the slopes of the relationship between those who were reactive and those who were non-reactive (Fig. 2).

### VVCR_m_ and VVCR_s_

**Fig. 3:**
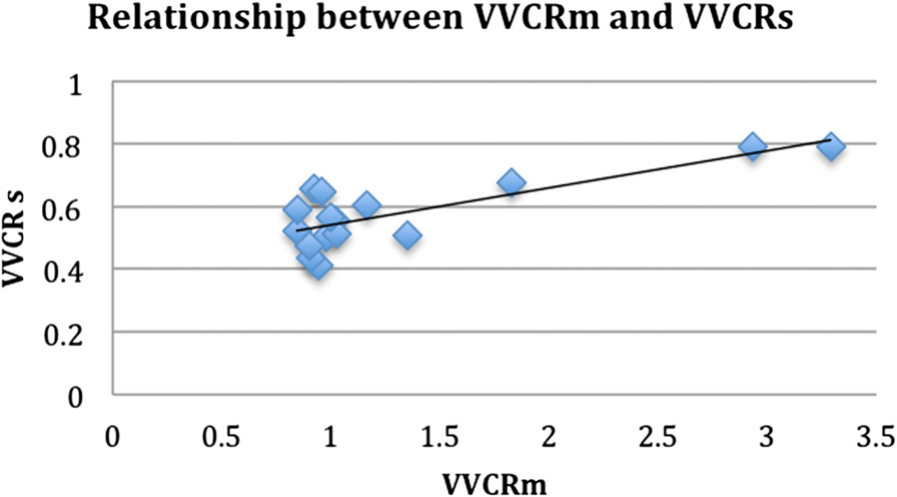
Scatterplot showing a positive linear relationship between ventricular vascular coupling ratio obtained by MRI compared to that obtained by single beat method

VVCR_s_ ranged from 1.25 to 2.4. VVCR_m_ was found to be significantly correlated with VVCR_s_ (r = 0.79, CI 0.48–0.99, p <0.0001) (Fig. 3). Heart rates were not significantly different during the catheterization compared to the MRI (92.2 bpm ± 26.8 vs 89.2 bpm ± 24.2, respectively; *p* = 0.7).

### VVCR and clinical outcome

**Fig. 4:**
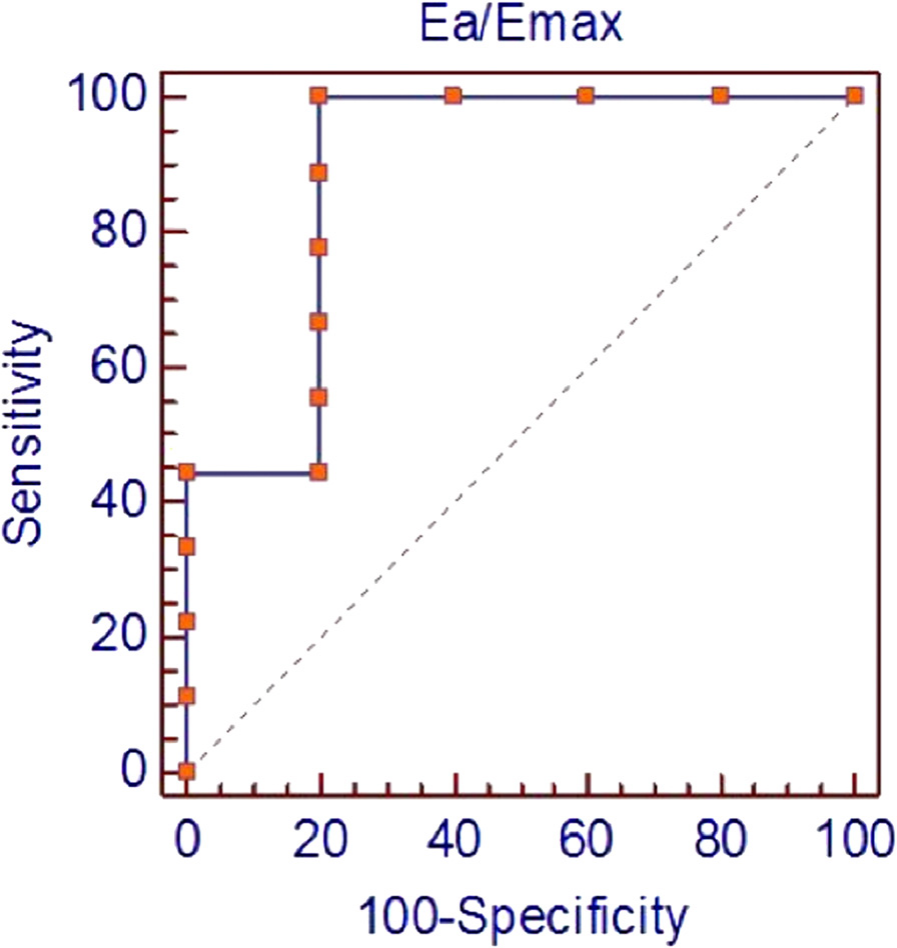
Receiver operating characteristic curve. Receiver operating characteristic curve demonstrates an optimal threshold ventricular-vascular coupling ratio (VVCR) of 0.85. Using this criterion, VVCR is associated with a sensitivity of 100 % and a specificity of 80 % in determining pulmonary reactivity

Receiver operating characteristic curve analysis (Figs. 3 and 4) revealed high accuracy of the VVCR_m_ in determining vascular reactivity. VVCR_m_ of 0.85 had a sensitivity of 100 % and a specificity of 80 %. The area under the curve is 0.89 (*p* = 0.008), suggesting good discrimination between those who were and were not reactive.

Table 2 describes the demographic features as well as the MRI data between subjects who had acute vascular reactivity. Subjects who showed positive reactivity were younger, with lower RVEDVi, as well as VVCR_m_. In addition, the median WHO-FC was 2 for the reactive subjects, and 3 for the non-reactive subjects. PVRi was not statistically significantly different, although the mean PVRi among the non-reactive subjects was more than double that of the reactive subjects. Similarly, although the length of disease was not statistically different between the two groups, the mean duration among the non-reactive subjects was almost twice that of the reactive subjects.

**Table 2:** Demographic data for reactive versus non-reactive subjects

Demographic data between non-reactive and reactive subjects			
	Non-reactive (*n* = 6)	Reactive (*n* = 8)	*p*-value
Age (years)	17 ± 4.1	8.5 ± 5.1	0.006
Female:male	4:2	4:4	
Length of disease (years)	10.3 ± 6	5.7 ± 3.8	0.1
Pulmonary hypertension classification			
1. Pulmonary arterial hypertension	6	6	
1.1 Idiopathic	1	3	
1.3 Drug and toxin-induced		1	
1.4.1 Connective tissue disorder	1		
1.4.4 Congenital heart disease	3	2	
1.4.5 Pulmonary hypertension from schistosomiasis	1		
3. Pulmonary hypertension due to lung disease		2	
PVRi (Woods unit x m2)	13.8 ± 11	6.1 ± 2.7	0.08
RVEDVi (ml/m2)	162 ± 61	100 ± 25	0.02
RVEF (%)	40 ± 14	50 ± 2.9	0.07
VVCR_m_	1.8 ± 1.0	1.0 ± 0.11	0.04

*Abbreviations:* *PVRi* pulmonary vascular resistance indexed, *RVEDVi* right ventricular end-diastolic volume indexed, *RVEF* right ventricular ejection fraction

## Discussion

In this paper, we have shown that the measurement of VVCR by CMR data in children is feasible. Furthermore, our study shows that VVCR derived from CMR data correlates with VVCR derived from the single beat estimation from catheterization-derived pressure tracings. The most intriguing finding here is the potential of noninvasively derived VVCR in predicting vasoreactivity. Acute vasoreactivity have been shown to independently predict transplant-free survival [21].

In the normal RV-PA axis, VVCR values range from 0.5 to 1.0, with the optimal ratio being 0.5 to 0.7 [22]. VVCR is significantly higher in the presence of PH.5 Fourie et al showed in invasive swine models that the RV at resting state operates at maximal efficiency when E_es_ > E_a_. In this state, the maximum stroke work is accomplished by minimum oxygen consumption [9]. The authors surmise that the RV maintains its reserve and contractility until E_a_ > E_es_ and stroke volume begins to fall. The concept of ventricular vascular coupling is essential to understanding PH, since this disease exemplifies the breakdown of the complex interaction between the vasculature and ventricle, ultimately resulting in RV failure. However, in clinical medicine, PVR continues to be primary parameter in which PH state is accessed. This is due to the labor-intensive, invasive nature of determining VVCR by establishing pressure-volume loops. Sanz proposed a non-invasive approach to determine VVCR based on CMR data [10]. Soon after this publication, Trip et al. contended that the assumption of a negligible volume at zero pressure, does not accurately assess E_es_ when using the Sanz’ approach [11]. Although we agree that the Sanz approach based on only CMR data may not be accurate, the benefit of a non-invasive approach to estimate the VVCR is an extremely valuable tool to serially follow PH patients over the course of their lifetime. This is particularly vital in our pediatric population since CMR avoids exposure to repeated radiation and decreases the need for general anesthesia. In Sanz’s data, there is a curvilinear relationship between VVCR_m_ and PVR. In contrast, our data shows a linear relationship. This may simply due to our small patient population. Both Sanz’s and our data covers a large range of PVRi, but with a significant sample difference of 150 in Sanz’s study compared to our 17 subjects.

In our study, the differences between the reactive and non-reactive subjects are significant. Not surprisingly, the PVRi trended towards higher values, and the RVEDVi and VVCR_m_ were higher among the non-reactive subjects. The median WHO-FC was also higher in this group. These parameters indicate a more severe disease status among the non-reactive children and thus, points to movement towards an uncoupled state. The non-reactive group is also significantly older with a trend towards having PH diagnosis for a longer time period, reflecting the progressive nature of PH. The slope of the line for the reactive patients is smaller than for non-reactive. This suggests that RV functional reserve is relatively preserved. Therefore, these children are able to accommodate an increase in afterload. However, the large slope in the non-reactive patients suggests that they are in the maladaptive remodeling phase and can no longer accommodate an increase in afterload. Future studies of the pediatric population and breakdown of etiologies will enhance the understanding of PH phenotypes.

VVCR_m_ is related to RV ejection fraction [10], which has been shown to predict mortality in adults [21, 23, 24]. However, there is currently no data in children on the relationship between RV ejection fraction and outcomes, catheterization data, or VVCR by the single beat method. Furthermore, while RV ejection fraction describes only ventricular systolic function, VVCR describes both the status of the vasculature and the ventricular efficiency in response to the changing afterload. Therefore, VVCR, and not RV ejection fraction, can discriminate changes in myocardial performance versus arterial load.

Limitations of this study include those inherent to a retrospective study. While the separation of 14 days between cardiac catheterization and CMR is longer than ideal, the infrequency of these two procedures being performed during a close time period for clinical care necessitated expanding our time frame; albeit 15/17 of the subjects had the procedures performed within 48 h of one another. We also recognize that the same patient may have had the cardiac catheterization performed under anesthesia while being awake for the CMR, which potentially can change the hemodynamic state. However, this is unavoidable since most pediatric catheterization at our institution is performed under general anesthesia. Anesthesia, on the other hand, is avoided for CMR if possible for children over the age of 8 years and may be a better reflection of the cardiovascular function of the subject at baseline. Two of the subjects had greater than mild tricuspid regurgitation. The additional volume from the tricuspid regurgitation would impact the RVEDVi, and thus, also impact the VVCR_m._ Moving forward, this factor must be teased out in order to understand the impact of tricuspid regurgitation in the progression of PH. The subject group in this study is small and is a significant limitation to the interpretation of this data. The data presented here is a preliminary look at the feasibility of using CMR in estimating VVCR in children with PH and to compare this approach to a more standard approach using cardiac catheterization. A larger subject population with data collected from multiple pediatric centers would be required to achieve adequate power. That, however, does not negate the importance of the findings in this study and is the stepping stone to posing the question for clinicians: can serial catheterization in children with PH be avoided if non-invasive imaging can provide adequate cardiovascular data for the management of these patients? And if so, this can potentially open the door for facilitating research in a vulnerable population that is unique from the adult population in whom the majority of PH research has been done.

## Conclusion

Measurement of VVCR by noninvasive means in pediatrics is feasible. Pulmonary vascular non-reactivity may contribute to ventricular-vascular decoupling in severe PH. Therapeutic intervention to maintain a low vascular afterload in reactive patients, may preserve right ventricular functional reserve and delay the onset of RV-PA decoupling. Use of VVCR may have significant prognostic implication.

## Abbreviations

CMR: Cardiovascular magnetic resonance
E_a_: Index of arterial load and right ventricular maximal end-systolic elastance
E_es_: Index of contractility
mPAP: Mean pulmonary artery pressure
PCWP: Pulmonary capillary wedge pressure
PH: Pulmonary hypertension
P_max_: Pressure at isovolumic contraction
PVR: Pulmonary vascular resistance
RV: Right ventricle
VVCR_m_: Ventricular-vascular coupling ratio by cardiac magnetic resonance
VVCR_s_: Ventricular-vascular coupling ratio by single beat estimation
